# Population Kidney Health. A New Paradigm for Chronic Kidney Disease Management

**DOI:** 10.3390/ijerph18136786

**Published:** 2021-06-24

**Authors:** Rafael Burgos-Calderón, Santos Ángel Depine, Gustavo Aroca-Martínez

**Affiliations:** 1 Medical Science Campus, Universidad de Puerto Rico, San Juan 00936-5067, Puerto Rico; rafaelburgosc@gmail.com; 2 School of Health Sciences, Universidad Simón Bolívar, Barranquilla 80001, Colombia; garoca1@hotmail.com; 3 Confederación de Asociaciones de Diálisis de la República Argentina, Buenos Aires C1053ABW, Argentina

**Keywords:** kidney health, population health, social determinants, sociopolitical context, environment, advocacy, interstitial nephritis, conservative care, dialysis, funding, kidney failure

## Abstract

Statistical data extracted from national databases demonstrate a continuous growth in the incidence and prevalence of chronic kidney disease (CKD) and the ineffectiveness of current policies and strategies based on individual risk factors to reduce them, as well as their mortality and costs. Some innovative programs, telemedicine and government interest in the prevention of CKD did not facilitate timely access to care, continuing the increased demand for dialysis and transplants, high morbidity and long-term disability. In contrast, new forms of kidney disease of unknown etiology affected populations in developing countries and underrepresented minorities, who face socioeconomic and cultural disadvantages. With this background, our objective was to analyze in the existing literature the effects of social determinants in CKD, concluding that it is necessary to strengthen current kidney health strategies, designing in a transdisciplinary way, a model that considers demographic characteristics integrated into individual risk factors and risk factors population, incorporating the population health perspective in public health policies to improve results in kidney health care, since CKD continues to be an important and growing contributor to chronic diseases.

## 1. Introduction

In public health, chronic diseases including chronic kidney disease have become a problem with difficult solutions, given the multiple causes that are linked to them. Not only are there biological factors, either those of genetic origin, or derived from people’s lifestyles, but also there are those factors that are beyond individual control: access to certain basic conditions or rights that are framed within human rights, education, basic sanitation, drinking water, decent housing, safe work, etc. The objectives of this review and subsequent proposal are to identify the magnitude of the problem, the current strategies that are applied in the current programs, their results and subsequently establish an integrating conceptual framework that generates a continuum, including individual risk factors and population risk factors, which can be applied in a practical way in public health interventions that improve how people are born, live, develop and die. Chronic kidney disease (CKD) is a serious public health problem and a major economic burden worldwide [1]. Currently, strategies to prevent and control CKD are primarily geared towards high-risk groups; however, the programs that have been implemented have failed to control the global increase in cases or to reduce the indirect consequences on the quality of life of affected individuals. Global data published in 2017 demonstrated that 1.2 million individuals died of CKD with an increase in the global mortality rate of 41.5% in the 1990–2017 period for all ages, representing the 12th leading cause of death worldwide in 2017 as opposed to 1990, when it occupied 17th place. The overall prevalence of CKD in all ages increased by 29.3%, resulting in a loss of 35.8 million disability-adjusted life years (DALYs). The major CKD burden was concentrated in the three lowest quintiles of the sociodemographic index (SDI), although all SDI quintiles showed a net increase in the absolute number of DALYs attributable to CKD from 1990 to 2017, which can be explained by population growth and aging. Moreover, as a secondary impact, there were 1.4 million deaths related to cardiovascular disease (CVD), and 25.3 million DALYs from CVD were attributable to kidney failure [2]. There is evidence that poverty is one of the conditioning factors for getting sick because it does not facilitate the development of healthy behaviors, hinders access to health care, and the environment where poor populations live often coexists with conditions of environmental toxicity and/or aqueous contamination with nephrotoxic agents such as Pb, Cd and As [3,4]. There are vulnerable populations in many parts of the globe with access to low resources, among which racial and ethnic minorities and indigenous peoples, in which detection campaigns have shown a high burden of kidney disease that usually remains hidden, without treatment and free to deteriorate. In many countries identified according to their gross domestic product as low or middle income, local factors of greater vulnerability are added to population groups with little possibility of demanding health improvements, for example, rural workers, the elderly and religious minorities, etc. [5] New kidney disease conditioning factors of unknown etiology have appeared. One example is the epidemic of nephropathy in agricultural communities, renamed CKD of nontraditional causes, in Central America, Sri Lanka and India, with high mortality rates in certain communities among individuals under the age of 60 and oversubscribed health systems. This is a form of chronic interstitial nephritis of multicausal origin whether it be by thermal stress, specific pesticides, genetic factors, infectious agents or social aspects [6]. Renal aggressors, facilitated by the inhospitable environment in which vulnerable individuals work, highlight the multicausal aspect of disease development, facilitated by existing inequalities in Latin America and the Caribbean [7]. If we begin with the proposal of measuring, analyzing and monitoring social inequalities in health, we are accepting that the social position of the individual is at the undisputed center of a broad conceptualization of health because it would allow the measurement of the real impact of population risk factors in relation to the possibility of getting individually sick. However, these individual actions would not be sufficient; there are extensive demonstrations that in CKD it is necessary to integrate population risk factors, included in the distal health determinants, with proximal determinants responsible for the biological context of health-disease, and their risk factors, assuming, a priori, that the summary of all risk factors, biological and population, are the primary contributors to the maintenance and growth of the incidence and prevalence of CKD in the screened populations [8]. Surprisingly, there is considerable evidence of the unequal burden of renal disease in communities which, at first sight, would appear to be similar, with adequate and similar healthcare structures and systems, even observing local variations in the predominance of one sex over the other [9]. These differences in prevalence are difficult to explain solely based on biological reasons, thus requiring an effort to identify and understand the causes of such differences, which are usually observed even within the same country [10]. It is well known and accepted that renal disease is still a global public health problem, thus showing a growing trend in more vulnerable populations and countries [11]. A meta-analysis reported that the prevalence of CKD worldwide outweighs that of diabetes, its primary cause, thus reaching 13.4% for stages 1 to 5, and 10.6% for stages 3 to 5, while the figure for diabetes was 8.2% [12]. The differential burden based on sex, most often impacting women, is inverted in certain communities, and it has not been possible to explain these differences [9,13,14]. Industrialized countries show higher prevalence rates than developing countries, although it is estimated that the growth of the number of inhabitants in underdeveloped areas is higher, and more populations will prematurely age; therefore, one can suppose that these populations experience difficulties accessing healthcare [15]. If the growth of chronic diseases, including CKD, is not controlled, this growing trend is of considerable concern for the sustainability of healthcare systems, in addition to the continued growth of the world’s population. Starting with a global population of 7.6 billion inhabitants, it is estimated that the number will grow to 8.6 billion by 2030, 9.8 billion by 2050, and 11.2 billion by 2100 [16]. Moreover, life expectancy is increasing, from 65 years for men and 69 for women in 2005, to 69 for men and 73 for women in 2015, with an older population with more disease burden demanding more healthcare [17], and more years lost to disability [18]. There are no concrete answers to the question of what is the underlying cause of the observed inequalities, which are replicated in many countries and regions. Evidence demonstrates that what has been done to date has not been sufficient to control the high incidence of kidney disease nor to understand its territorial differences. One possibility is the establishment of expanded surveillance systems, including for acute renal failure, which is a risk factor for CKD, and which currently shows a high incidence. Conspicuously, public health agendas have not included CKD in their priorities despite its increasing impact on national public expenditure and the economy of families with loss of quality of life for the sick expressed in a growing number of years of life lost to disability [19]. Moreover, the Global Action Plan for the Prevention and Control of Noncommunicable Diseases (NCDs), promoted by the World Health Organization in 2013, and its counterpart in the Americas, the Pan-American Health Organization, have not included CKDs within NCDs, unlike CVDs, cancer, chronic respiratory diseases and diabetes, which have been included [20]. World Bank data provide troubling information on how countries have assessed their activities in relation to CKD (Table 1) [21].

The provision of adequate resources and a workforce to establish and maintain surveillance systems (e.g., screening and registration programs) is essential and requires substantial investment as well as preserving the principles of ethics in budget allocation [22]. Currently, researchers have focused on genetic, environmental, sociodemographic and clinical factors of kidney disease. Even racial and ethnic minorities are disproportionately affected by advanced and progressive kidney disease and its neglected risk factors [23]. A comprehensive model for measuring social health inequities, called the Health Equity Measurement Framework (HEMF), has recently been published, and includes the socioeconomic, cultural and political context, health policies, social stratification, social stratification, material and social circumstances, the environment, biological factors, health-related behaviors and beliefs, stress, quality of care, and the use of healthcare systems [24]. It seems that, despite the letters of intent signed by the highest representatives of the countries, achieving equality is part of the utopias, as well as reducing inequality between the populations of different countries, regions or even within the same countries; e.g., the World Conference on Social Determinants of Health, [25] held in Rio de Janeiro, Brazil, between October 19 and 21 of 2011, entitled “All for Equity”, which culminated in a Declaration expressing “the political commitment for the implementation of a social determinants of health approach to reduce health inequities and to achieve other global priorities”, and committing to implement actions aimed at:

- Adopting better governance for health and development;
- Promoting participation in policymaking and implementation;
- Further reorienting the health sector towards reducing health inequities;
- Strengthening global governance and collaboration;
- Monitoring progress and increasing accountability;
- Concreting a call for global action.

**Figure 1:**
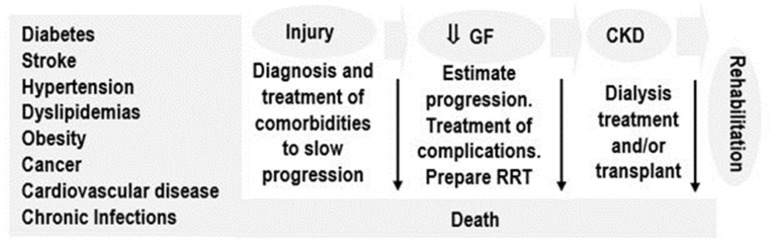
The continuum of renal disease focusing on individual risk factors. Produced by the authors.

No major progress has been made since then; it is in this context of inequities and inequalities where CKD finds the most fertile ground for its development and continued growth and where the most unprotected people are affected. Current strategies have primarily focused on addressing individual risk factors; however, evidence shows that they have not yielded the expected results, i.e., decreased incidence, prevalence and morbidity of CKD (Figure 1).

**Figure 2:**
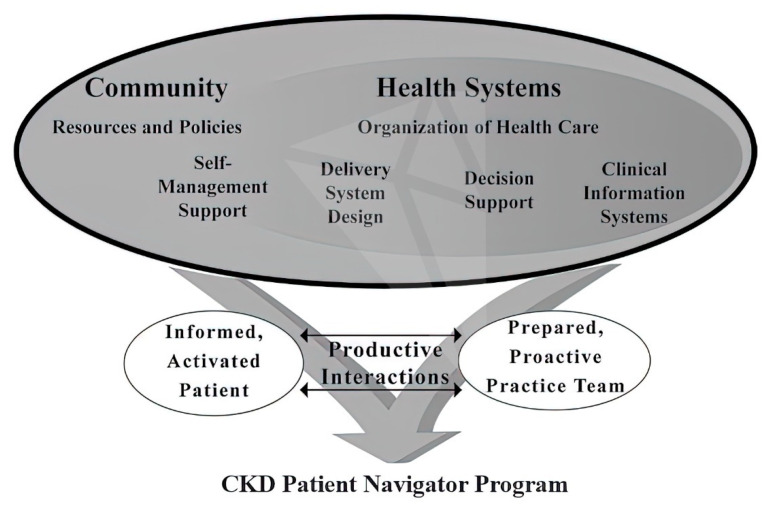
Chronic care model and its relationship with the CKD Patient Navigator Program. Source: Wagner et al. [36,39].

Scientific advances in the healthcare field have increased life expectancy and the quality of life of many individuals around the world. However, these improved living conditions coexist with situations of social distress and great cultural and economic deficiencies, which entails the possibility of sickness and death from diseases that could be controlled under situations of equity and justice. It has always been considered that the biomedical aspect is not sufficient; however, not many effective interventions have been proposed to minimize the consequences of disparities in health coverage in vulnerable populations, with low literacy and education levels, low economic income, poor health coverage/lack of accessibility, and ethnic and/or racial issues associated with disadvantage. While the emphasis has been on highlighting the importance of social determinants of health [26], not much has been done in most undeveloped countries, and the greatest economic and programmatic efforts still aim at strengthening hospital facilities and subsidizing the supply side rather than the real demand from populations rather than increasing primary health care and the first levels of care to support the most complex levels [27]. Social and cultural factors influencing causes, as well as the manner or possibilities in which persons use or receive healthcare are increasingly significant. The International Society of Nephrology is generating great global hope, as it has planned an ambitious five- to ten-year master plan to comprehensively address many of these inequities in kidney health care. For this, it has developed a strategic plan for the comprehensive care of patients with kidney failure, which is transdisciplinary and focused on monitoring, dialysis, resources, transplantation, and conservative kidney management [28]. Precision medicine is now considerably valued because sequencing analyses have been able to identify numerous genetic variants related to specific kidney diseases, usually those manifesting early in life and caused by single gene mutations [29]. However, significant numbers of kidney diseases are genetically and phenotypically complex; it is necessary to understand how those genetic particularities interact with the environment, thus making it possible to classify CKD into molecularly defined subgroups, which would lead to specific therapeutic interventions [30,31,32]. However, these considerable efforts of genomic identification are lost in large population masses, even if they are performed, because of the social conditions in which they are immersed. In a study of 500 vulnerable adults, a possible pathogenic variant was detected in ten participants (2%). The problems were the time that elapsed until results were reported (582 ± 53 days), as well as difficulties contacting the subjects, loss of appointments, illiteracy, limited access of participants, lack of knowledge about their health coverage, little family background information, and, in some cases, situations of distress because of recent traumatic processes [33]. There is evidence that precision personalized medicine, despite the advances made in the detection of the genomic aspects of disease, will not be able, on its own, to overcome the obstacles created by health disparities and inequalities. In certain chronic high-impact pathologies, it is common to use computer or communication tools to improve access and the care delivery process. This has demonstrated great utility and good results improving the holistic health of patients and family members, including, e.g., programs involving personalized contact with patients which are being of great importance in the COVID-19 pandemic [34,35]. Its beginnings were based on oncological practice, studying the relationship between cancer, survival and social conditions, which demonstrated that social position, economic status, culture and the environment were critical determinants of whether a person would develop and/or survive cancer, and directly impacted the quality of life of survivors. These were pioneering studies for implementing a patient navigation program to alleviate these unfavorable situations [36]. Based on this knowledge, patient navigation programs have been implemented to improve healthcare. Initially, they were implemented in oncological programs, for cancer patients from vulnerable populations, and offered personalized logistical and emotional support through a continuous process of care from disease detection to diagnostic evaluation and treatment. Their purpose is to help patients overcome barriers to healthcare access, facilitating quality care, without confronting their culture. Workers, called navigators, assist patients in finding solutions for their coverage, facilitate appointment coordination, support understanding through linguistic literacy assistance, and can provide advice on health coverage rights, ultimately providing educational elements for self-care [37]. This indicates a requirement for academic relearning of the transversal generic competencies of healthcare teams. This has been the conceptual framework for the recent implementation of the previously mentioned CKD patient navigator program, which strives to make each patient and their healthcare team proactive and better prepared to respond as per the guidelines and principles of the chronic healthcare model, resulting in informed and active patients [38]. (Figure 2).

**Figure 3:**
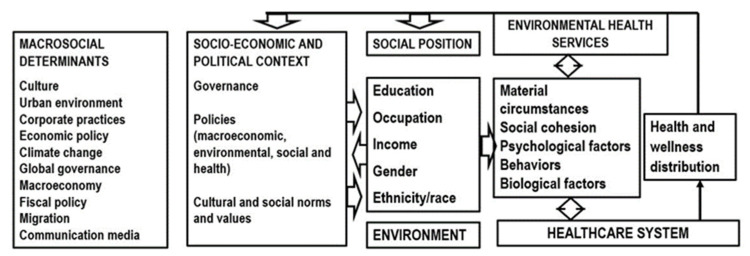
Conceptual framework of the social determinants of health. Source: Mújica and Moreno [43].

Despite efforts that have been made, the CKD incidence has not decreased. Therefore, it is essential to transform the prevailing biomedical model, based on risk factors and individual behavior, into one that explicitly includes the risk factors of the population, which constitute the social or distal determinants of health [22,40], and which condition individuals coming from poor and/or vulnerable populations to develop disease, including noncommunicable chronic diseases, which are responsible for high morbidity and mortality rates and disability in large human groups from various forgotten regions of the planet [6]. The differences between public health and population health have been conceptually defined; despite these differences, each of them subsumes the other. These definitions often agree in defining public health as the science whose objective is to protect and improve the health of communities through policy recommendations, education, promotion through communication of healthy habits, and research for the prevention, early detection and treatment of diseases and injuries. It is a collective action, with a transdisciplinary approach, with full participation of society, regardless their educational, professional or academic training. However, population health generates opportunities such that social systems and structures related to holistic health and agencies and medical organizations work together to improve the health outcomes of the communities they serve, thus effectively controlling the risk factors of a population that impacts individual health. In 2003, Kindig and Stoddart defined population health as “the health outcomes of a group of individuals, including the distribution of such outcomes within the group, related to the social determinants detected within the assessed groups”, understanding by group not only geographical regions, nations or entire communities but also ethnic minorities, disabled persons, or individuals deprived of their freedom, among other studied groups [41]. Colmenares and Eslava-Schmalbach [42] emphasized the concept of population health, uniting the causes of individual health (genetic, cultural, and microenvironmental), with those of population health (environmental, contextual, economic, political, and psychosocial), and occupational causes. These definitions incorporate health determinants into public health components; since CKD is a major exponent of chronic diseases in continuous growth, it is necessary to generate innovative proposals. Therefore, we discuss our proposal for expanding existing models of kidney health, tending towards a new model of population kidney health, contributing to research and detection of traditional risk factors, those population risk factors which, although not related in a first analysis with biological issues, end up impacting individual health. As an antecedent of great importance, the World Health Organization’s Commission on Social Determinants of Health (CSDH) proposed a model that conceptualizes the impact of social determinants of health on health care systems and their impact on equity in health and well-being, a scheme that was expanded by Mujica and Moreno in their work entitled “From Rhetoric to Action: Measuring Health Inequalities to ‘Leave No-one Behind,” in which they proposed to institutionalize the measurement, analysis and monitoring of social inequalities in health to act on the social and environmental determinants of health and achieve sustainable development, universal health and social justice for all [43]. (Figure 3).

## 2. Materials and Methods

A review of published work related to the topic of social determinants of chronic kidney disease was conducted. The following research questions were specified to guide the identification of relevant material before beginning the review work: Is there a link between social determinants and disparities in CKD rates and outcomes? Are social determinants being incorporated into current policies and approaches for the management of CKD? Based on these questions, a search strategy was built which consisted of doing a preliminary search in major scientific databases including MEDLINE/Ovid, PubMed, and Google Scholar using the search terms Chronic Kidney Disease, renal disease, renal transplant, and dialysis in combination with any of following key terms: social determinants, socioeconomic status, gender, race, social inequalities, social inequities, social disparities, low-income, poverty, developing countries, rural communities, and other similar search terms. Publications from well-known experts that have conducted research on the effects of social determinants on CKD were also searched. Titles and abstracts of records identified by the searches were assessed by the three authors independently. Duplicated, abstract-only papers and material without the available full text were excluded. Full-text copies of material that could potentially meet the review inclusion criteria were assessed against the inclusion criteria by the authors. To meet these inclusion criteria and be eligible for review, studies had to describe and report on the effects of one or multiple social determinants on CKD outcomes. Randomized and nonrandomized controlled trials, observational studies, and surveys of the literature were eligible. Policy papers that document the impact of social inequalities on CKD outcomes, or describe public health models to combat CKD were also eligible. The findings from individual studies were summarized. For each study, relevant social components, and their possible pathways to influence CKD outcomes were identified. A conceptual framework of the population kidney health model was created, which is a synthesis of these theoretical components identified across the literature.

## 3. Results

### 3.1. Results

**Figure 4:**
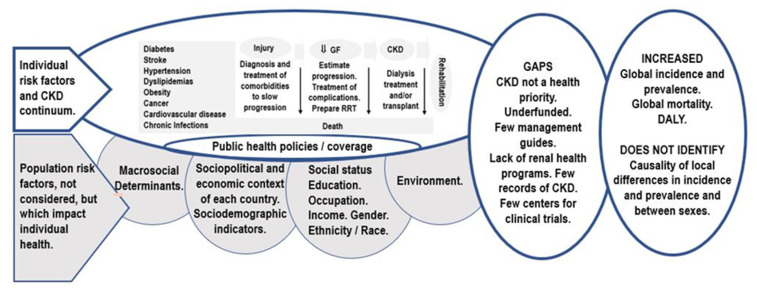
Biomedical model of CKD, which does not include population risk factors. Source: Authors’ own creation.

**Figure 5:**
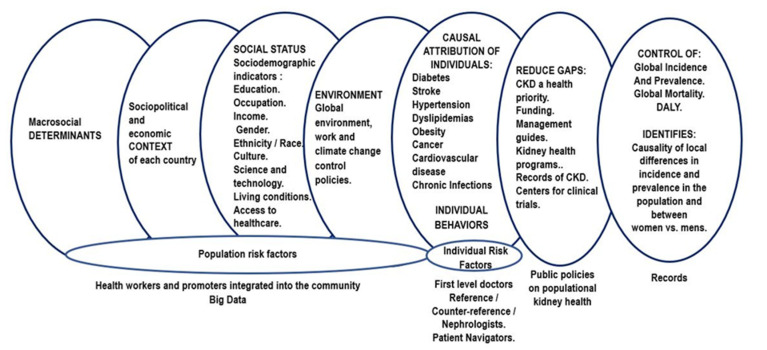
Conceptual framework of the population kidney health model. Source: Authors’ own creation.

There is an abundance of evidence showing the relationships between social and environmental factors and CKD. Across the literature reviewed, country-level socioeconomic factors like health expenditure [7,18], development level [17], and the size of the kidney care workforce [1,11,21,22], individual-level sociodemographic characteristics like gender [9,12,13,14,27], education [2,34], household income [33,34,44,45], race and ethnicity [8,23,29], as well as environmental factors like exposure to agrochemicals and high-temperatures [6,44], were identified as consequential to CKD outcomes. However, even though there is an increasing number of studies indicating the relevance of these social determinants, our review showed that public health policies for CKD management are still predominantly focused on health-related individual risk factors [8,43]. This problem is summarized in Figure 4. Moreover, the review exposed the need to create a model that integrates social determinants and individual risk factors. The creation of an integrated model to measure social inequalities in health, coupled with the recognition of CKD as a priority in public health, will allow for the inclusion of relevant and targeted programs in governmental budgets, such as the expansion of data collection initiatives, the creation of public health, education, and research programs focused on CKD management and prevention, as well as strategies to promote the adequate distribution of medication and CKD services to vulnerable populations, all of which will improve health outcomes in CKD, particularly in disadvantaged groups, while also contributing to the advancement of Sustainable Development Goals set by the United Nations. In the section below (Figure 5) we present our conceptual framework of the population kidney health model, which is a synthesis of the theoretical components from the reviewed social determinants of CKD literature.

### 3.2. Discussion

This way of approaching the health-disease-care-rehabilitation process is the biomedical model par excellence, in which not all the population risk factor components that impact health are included (grey shading), leaving many persons in unfavorable and vulnerable conditions prone to falling ill, for example, with kidney disease, and without early and adequate medical care, resulting in permanent CKD, or death (Figure 4).

For this reason, it is necessary to generate a paradigmatic change, expanding the approach to the problem of CKD through a model that analyzes the variables of inequity and inequality locally, establishing objectively verifiable indicators that allow the measurement of the impact on the kidney health of populations, with special emphasis on the most vulnerable and disadvantaged ones [44,45]. The proposal presented here is that of a model of kidney health, including an analysis of public health policies and levels and logistics of coverage for their improvement, and which integrates other variables which, operationalized in the field, allow an effective integration of individual risk factors with the risk factors of the population, including the transdisciplinary participation of social actors recognized as community leaders. This model, which adds the word “population” to the kidney health statement, can generate health policies that include the distal determinants of health, the social determinants, which combine with the proximal or biological determinants, such that both are holistically articulated and addressed together to reduce or control the burden of kidney disease, which reduces the high costs for health systems and families and contributes to improving the quality of life of the sick and promoting health care. In summary, the population kidney health model should articulate individual risk factors with the risk factors of the population that impact individual health, to control the incidence and prevalence of CKD and decrease the overall severity and years of life lost to disability, as measured by the DALY indicator. Its importance lies on the possibility of using indicators derived from previous data collection instruments, such as the Health Equity Measurement Framework and the National Nutrition and Health Surveys, which incorporate research variables such as the region where surveyed subjects live, as well as their sex, age group, educational level, health coverage, the quintile of household income per consumer unit, the type of educational establishment attended, the presence of overweight, obesity or excess weight, and dietary patterns, including the renal function estimated with a formula after measuring plasma creatinine with the kinetic Jaffé method, and renal damage determined by the relationship between albumin and creatinine in an isolated morning urine sample. The sum of all these variables and indicators will allow for determining the current risk factors, at both individual and population levels, to explain the causes of the differences in incidence and prevalence that are usually observed among apparently similar populations, as well as the fluctuating variation in the load of the disease according to localities and between sexes (Figure 5). Health agents, health promoters integrated into the community, including community nurses, deserve special attention. It is necessary to use big data technology that analyzes and interprets large volumes of data, to enable, through correlations, the best decision-making and obtain the best results, in the fields in which it is applied, including public health.

With wide access to social networks, a communication complication has arisen. Called an infodemic, it is defined as an overabundance of information, often incorrect and potentially fatal for patients with chronic diseases, for example, chronic kidney disease, which is considered at the epidemic level. Alerted by this situation, the WHO has installed a new information science called infodemiology, to adequately manage all the information of popular scope to raise awareness in the population about the best practices of self-care and self-preservation of kidney function. Population kidney health must include early, strategies derived from infodemiology [46]. We believe that public health requires to change its usual model of disease care, moving toward health policies that contain social development factors that impact individual health but in turn address the serious problem posed by CKD in all the countries of the world [2]. To this end, we interpret that an action research methodology, which promotes the performance of sociocritical or socioconstructivist studies, will be able to identify possible structural failures in the social functioning dynamics of existing primary, secondary and tertiary prevention programs, and improve them to achieve the objectives for which they were created. This, in the case of kidney health, presupposes an efficient control of the disease in its different development stages and knows in advance that the social position of the members of society and the habitat in which they live are fundamental when accessing healthcare services. The latter point can be exemplified considering the set of negative social conditions observed in many countries, regions or communities. An example of this is what happens in ethnic, religious or linguistic minorities, as in many indigenous, Afro-Latin and Afro-Caribbean communities, in which their poor socioeconomic status adds to their linguistic communication difficulties, their poor education, and their lack of accessibility to healthy eating, with excess consumption of sugary fats and drinks, which prematurely increase the risk of getting sick and dying, especially from chronic diseases, including hypertension, diabetes and kidney disease [23,47,48,49,50]. In women of childbearing potential, poor nutrition adds another risk factor of low-weight newborns with renal maturation deficits [51]. In relation to the environment, there is evidence linking agrochemical toxics with CKD of unknown cause in agricultural communities, together with other concurrent causes such as dehydration and heat stress during rural labor without adequate national worker protection regulations [52]. Among known risk factors, hypertension remains a primary risk factor associated with CKD, present in >90% of individuals with advanced kidney disease, and being associated with disease progression [9,53]. It affects one billion people [54] worldwide; therefore, its strict control must include decreased consumption of dietary salt and preserved foods, forming a part of the set of healthy policies linked to health determinants. Acute kidney failure is linked to unfavorable conditions in certain countries and is associated with high morbidity and the possible evolution to chronicity in survivors; therefore, its prevention, identification and early treatment, in addition to health aspects, and like the rest of preventable diseases, have an ethical correlate in the context of human rights [55]. The delay in considering CKD as a priority in healthcare policies and the absence of global planning that includes the distal determinants of health could explain the deficiencies seen in accessibility to care in the implementation of kidney health programs in predialysis situations and in the training of specialized human resources, which are becoming increasingly scarce [18,22]. The lack of encouragement of young professionals to specialize in nephrology is very evident in the least developed countries [56], a situation similar to that reported in past decades [57], and it is essential to modify these issues to successfully address the control of CKD. To solve these complexities, it is necessary to articulate different successful public and private healthcare strategies, generating adequate articulations between the different systems and service sectors, acting within the framework of public population kidney health policies (Figure 5), giving visibility to health workers and health promoters for the local and family detection of population risk factors, and relying on georeferencing [58,59], and new big data computer technologies. The next step following the detection of a sick person would be to facilitate their access to primary care physicians who, through specialized clinical guides and flowcharts, can expedite the reference and counter reference mechanisms required to refer the case to specialists, and with the patient navigator program act always as a support to patients. As a fundamental strategy for validating the entire process, the development and permanent updating of renal patient records in different disease stages, dialysis, and transplants will be encouraged, including the records of patients who abandon their treatment with their causes, if possible, to be proactive in similar situations, and to maintain the records of CKD patients under palliative care.

### 3.3. Research Limitations

The limitations of the evidence are linked to the cut-off moment of the bibliographic research. Additionally, in our review it is possible that there are biases derived from the authors who have published their experiences, including the great problem posed by the under-registration of patients due to the lack of reliable data at the international level, especially in countries with less resource capacity technological. However, in the analyzed bibliography there is a coincidence in the way in which social determinants are correlated with the possibility of health care, but, as these determinants are pointed out, little is being done in the field of public health to facilitate possible control strategies over these determinants. That is why we initially propose this conceptual framework of population kidney health, in order to motivate the use of information strategies and technologies in later stages that correlate epidemiological, social and individual factors, hoping to achieve the possible “control” of chronic kidney disease in the most vulnerable populations.

## 4. Conclusions

Due to the continuous growth of kidney disease in the world, CKD continues to be one of the main health problems for the governments of all countries. This is not only due to the demand for specialized technological and human resources for its treatment, but also to its high financial impact on health systems and the affected families. As their global costs are of great magnitude, they consume important percentages of the GDP of the countries. On the other hand, there are significant expenses derived from direct and indirect costs, linked to the disabilities that accompany the worsening of kidney function. In November of 2004, during the Controversies Conference on the Definition and Classification of Chronic Kidney Disease in Adults Worldwide, held in Amsterdam, Netherlands and organized by KDIGO (Kidney disease: Improving Global Outcomes), we discussed, as part of a proposal from the Latin American Society of Nephrology and Hypertension, the need to implement a kidney health model [60]. This model, globally or in its parts, was introduced to several Latin American countries, as expressed in the editorial publication of the journal Nature Clinical Practice Nephrology [61] in May 2008: “[…] a committee chaired by Santos Depine and Rafael Burgos Calderón has developed models of renal health that can be combined with the disease surveillance and prevention programs already functioning in some countries (Depine S and Calderón RB [2006] Ren Fail 28: 649–664). The committee has established official contact with 12 countries (Chile, Argentina, Uruguay, Venezuela, Colombia, Brazil, Paraguay, Bolivia, Mexico, Puerto Rico, Peru and Ecuador). As a result of these contacts, nine countries (Chile, Argentina, Uruguay, Venezuela, Colombia, Brazil, Paraguay, Bolivia and Mexico) have signed a memorandum of understanding to initiate renal disease prevention programs, and eight countries (Chile, Argentina, Uruguay, Venezuela, Colombia, Brazil, Paraguay and Puerto Rico) have introduced such programs. This model, expressed in kidney health programs, has been moderately successful. However, in most countries around the globe, detection programs have been implemented, the drawback of which is that these detections are usually carried out on the population that accesses the consultation, leaving large population conglomerates without the possibility of access to the system and therefore suffering from “hidden kidney disease”. The few registries available take as a reference the patients who have been detected, despite which, the data reveal a continuous growth in prevalence and incidence rates, which suggests that the usual forms of public health management, focused mainly on treating diseases, have not been totally effective in controlling the problem of CKD. We interpret that this means we are faced with the great challenge of evaluating new ways of managing chronic diseases, including CKD. This implies the need to reformulate the learning and knowledge acquired, unlearning to relearn different ways of providing possible solutions to the problem raised. Just as the dominant paradigm has focused on disease care, the time has come to observe the health-disease-care-rehabilitation process in a more holistic way, incorporating, in addition to the variables of biological determinants, those that are related to social determinants and that end up having an impact on people’s health, as was postulated by Mújica and Moreno in the PAHO [43]. Therefore, and based on the current biomedical model, we conclude that it is necessary to generate a paradigmatic change by operationalizing a model that we proposed, Population Kidney Health, which incorporates both social and biological variables, not only to facilitate the control of kidney disease, but also to enable greater accessibility of the people to the health system and the best available health care.

## Figures and Tables

**Table 1:** Chronic kidney disease gaps by World Bank country groups.

CKD Care	Low-Income Countries (%)	Lower-Middle Income Countries (%)	Upper-Middle Income Countries (%)	High-Income Countries (%)
Government recognition of CKD as a health priority	59	50	17	29
Government provides funding for all aspects of CKD care	13	21	40	53
Availability of CKD management and reference guidelines (international, national or regional)	46	73	83	97
Current existence of CKD detection programs	6	24	24	32
Availability of dialysis records	24	48	72	89
Availability of academic centers for the management of renal clinical trials	12	34	62	63

Source: Global Kidney Health Atlas. (CKD: chronic kidney disease).

## Data Availability

Not applicable.
